# Editorial: Nutrition and mood disorders

**DOI:** 10.3389/fnut.2025.1744366

**Published:** 2025-12-15

**Authors:** Shalini Mani, Samiksha Wasnik, Manisha Singh

**Affiliations:** 1 Department of Biotechnology, Jaypee Institute of Information Technology, Noida, India; 2 Independent Researcher, Gilroy, CA, United States; 3 Discipline of Pharmacy, Graduate School of Health, Faculty of Health, University of Technology Sydney, Sydney, NSW, Australia

**Keywords:** mood, nutrition, disorders, mental health, food

Mental and neurological conditions account for a significant proportion of the global disease burden, affecting hundreds of millions of people (1). Depression, anxiety, cognitive impairment, and stress disorders reduce quality of life and generate enormous social and economic costs (1). With the increase in the number of such problems, the intricate relationship between nutrition and mental health has garnered significant attention in recent years. Consequently, nutritional psychiatry is coming up as a new discipline, discovering the role of micronutrients, macronutrients, dietary habits, metabolic conditions, and nutritional awareness in mental health. Foods fortified with magnesium, folic acid, omega-3 fatty acids, and vitamin E are reported to support mental health and cognitive function by delivering necessary nutrients (2). Also, with continued studies in the area of nutritional psychiatry, the role of diet in the prevention and treatment of mental health is becoming more apparent (3, 4).

Following this increasing appreciation of the relationship between diet and mental health, this current Research Topic “*Nutrition and Mood Disorders*” of Frontiers in Nutrition presents a Research Topic of 12 articles, covering large-scale epidemiological studies, randomized trials, systematic reviews, as well as mechanistic studies, providing a comprehensive understanding of the intricate relationship between diet and mental health. These inputs include a variety of populations, cultural backgrounds, and scientific frameworks, but come together on a common consent that nutrition is a critical determinant of mental health and a potential therapeutic target. These 12 studies made the following important points:

Chu et al. investigated sex-specific variations in the relationship between dietary riboflavin (vitamin B2) consumption and depression or suicidal ideation within a substantial NHANES-based population (*n* = 29,466). The analysis revealed a non-linear inverse relationship evident exclusively below an intake threshold of approximately 1.4 mg/day. Their research showed that while contemplating the utilization of nutrients to enhance mental health, it is essential to take into account both the quantity ingested and the individual's sex.

Słupski et al. performed a systematic study to investigate the correlation between appetite-related indicators and clinical responses to ketamine treatment in mood disorders. The analysis revealed diverse methodologies among studies but underscored that alterations in appetite may act as possible indicators or predictors of ketamine efficacy, stressing the necessity for standardized methods and additional prospective research.

Salamanca-Sanabria et al. conducted a secondary study of clinical data to examine the impact of adherence to an Asian-adapted Mediterranean diet on depression and anxiety outcomes in women with metabolic dysfunction-associated steatotic liver disease. The results indicated that the modified food pattern might provide mental health advantages; nonetheless, the authors underscored the need for larger randomized controlled trials to validate causal relationships.

Xu et al. examined NHANES 2011–2014 data to evaluate the relationships among nocturia, depression, and cognitive performance in older individuals, along with the mediation influence of nutritional indicators. The findings demonstrated that nocturia was associated with elevated depression levels and worse cognitive performance, with serum albumin and hemoglobin partially moderating these relationships. The results suggest that enhancing nutritional status may alleviate the mental and cognitive difficulties associated with nocturia, especially in women.

Starck et al. conducted a pragmatic random trial involving nearly 1,000 free-living Australian adults to compare the effects of A1-protein-free milk with conventional milk. The study found that individuals who consumed A1-free milk experienced improvements in mood and subjective cognitive function, including reductions in anxiety, stress, sadness, and fatigue over 4 weeks. These findings suggest that variations in milk protein composition may influence emotional state and cognitive performance, though the authors emphasized the need for confirmatory studies to validate these preliminary results.

Gu et al. utilized CHARLS data to examine the correlation between the Lipid Accumulation Product (LAP) an indicator of visceral adiposity and the prevalence of depression in middle-aged and older Chinese men. The study revealed a notable U-shaped relationship, where both low and high LAP values were associated with an increased risk of depression, suggesting that a moderate amount of body fat may be beneficial for mood regulation. These findings highlight the complex interaction between body-fat distribution and emotional wellbeing.

Li and Lan examined NHANES data from 2007 to 2016, analyzing over 18,000 U.S. adults to assess the correlation between dietary lycopene consumption and depression. They discovered that increased lycopene intake was associated with a reduced likelihood of depression, although the benefits diminished at higher levels, forming a U-shaped relationship. The authors suggested that this association may involve antioxidant and anti-inflammatory pathways and advocated for longitudinal investigations to clarify causality.

Rana et al. evaluated observational and interventional research investigating the roles of natural sulfur-containing compounds, such as sulforaphane, taurine, allyl sulfides, hydrogen sulfide donors, and sulfated metabolites, in mental and neurological health. He highlighted evidence suggesting that consuming sulfur-rich foods may protect the brain, reduce inflammation, and inhibit oxidative stress, potentially benefiting conditions such as schizophrenia, depression, and anxiety. The study emphasized the neuroprotective and neurotransmission-modulating effects of these compounds while noting existing research gaps in translating these biochemical findings into therapeutic applications.

He et al. evaluated the correlation between blood uric acid concentrations and depression using NHANES data from more than 23,000 participants. The study found that individuals with higher uric acid levels were less likely to experience depression, particularly among older adults, individuals with heart disease, and certain ethnic groups. The authors also explored potential mechanisms involving oxidative stress and purinergic signaling and acknowledged the limitations of cross-sectional designs in establishing causality.

Ebrahimi et al. investigated the correlation between lifestyle risk scores—encompassing diet, physical activity, sleep, smoking habits, and social background factors—and mental health outcomes in overweight and obese Iranian women. The study found that higher composite lifestyle risk scores were associated with poorer mental health indicators, emphasizing the importance of addressing multiple lifestyle domains rather than isolated habits. These findings highlight the value of integrated lifestyle interventions in improving mood and overall wellbeing within this demographic.

Ghabashi examined the correlation between nutritional awareness and generalized anxiety disorder among young adults in Saudi Arabia. The cross-sectional study revealed that increased nutrition knowledge correlated with reduced GAD symptoms, indicating that improving nutritional education may support national preventative mental health initiatives in accordance with Saudi Vision 2030.

Lv et al. investigated the correlation between serum β-carotene concentrations and suicidal thoughts in adults utilizing cross-sectional data. The findings indicated an inverse correlation, with elevated β-carotene levels associated with less suicidal ideation. The authors suggested antioxidant level as a possible protective mechanism and recommended long-term studies to further investigate this relationship.

Collectively, the articles featured in this Research Topic underscore the intricate interplay between dietary components, nutritional biomarkers, and mental health outcomes. Future studies should adopt longitudinal and mechanistic designs, harmonize nutritional and psychological assessments, and explore sex- and culture-specific factors to translate nutritional psychiatry insights into effective preventive and therapeutic interventions.
